# COMPARING STROKE MORTALITY TRENDS IN RURAL AND URBAN AREAS DURING AND BEFORE THE COVID-19 PANDEMIC

**DOI:** 10.1093/geroni/igae098.3942

**Published:** 2024-12-31

**Authors:** Vivian Lin, Sasha DiVall, Mengyuan Cheng, Nasim Ferdows

**Affiliations:** Northeastern University, Boston, Massachusetts, United States; Northeastern University, Boston, Massachusetts, United States; Northeastern University, Boston, Massachusetts, United States; Northeastern University, Boston, Massachusetts, United States

## Abstract

COVID-19 has undeniably disrupted healthcare services, with many individuals advised to avoid hospital visits unless necessary. However, timely treatment following a stroke is critical to minimize damage or fatality, raising concerns about how these disruptions may have affected stroke outcomes. This study explores the association between COVID-19 and stroke mortality rates across different ruralities across a three-year period (2018-2020). Using data from the CDC WONDER database and County Health Rankings, we examined stroke mortality trends, adjusting for county-level characteristics. The study classified rurality using Rural-Urban Commuting Area codes from the Economic Research Service into three categories: urban, rural-adjacent to an urban area, and rural non-adjacent to an urban area, given the generally lower access to medical care in rural settings. Our analysis, which utilized data up to 2020—covering less than a full year of the COVID-19 pandemic—found no significant differences in stroke mortality rates across the different rurality levels. However, it is important to note that the limited data from the early stages of the pandemic may not fully capture the potential impact of COVID-19 on stroke mortality. As more recent data from 2021-2022 become available, we anticipate that a more comprehensive analysis could reveal different trends, particularly as the effects of prolonged lockdowns and healthcare disruptions become more evident. This research aims to provide a clearer understanding of stroke mortality trends during the pandemic, offering insights that may guide healthcare practices and policy decisions to ensure that all populations, especially those in rural areas, have access to necessary care.

